# Supplementary material for: Adaptive guidelines for the treatment of gonorrhea to increase the effective life span of antibiotics among men who have sex with men in the United States: A mathematical modeling study
Source: PLoS Med. 2020 Apr 3;17(4):e1003077. doi: 10.1371/journal.pmed.1003077 (PMC7122693; doi:10.1371/journal.pmed.1003077)
Supplement: S1 Text — (PDF) [file pmed.1003077.s001.pdf]

S1 Text: Additional Details on Model Structure, Calibration Procedure, and the Algorithm to Identify Adaptive Policies

S1 Additional model details

S1.1 Model population

We developed a stochastic compartmental model to simulate the transmission of gonorrhea among the MSM population of age 14 or older in the United States (Fig. S1). A meta-analysis of U.S. population-based surveys estimated the proportion of MSM among male 13 and older at 3.9% [1]. According to the 2015 U.S. Census, the size of the male population of age 14 and older is 125,092,000 [2]. Therefore, we approximate the MSM population of age 14 and older at 4,878,588. We assumed that an individual stays in the model for an average of 35 years (representing the period when an individual could be sexually active).

S1.2 Simulation approach

To construct the model, we introduce the following notation:

- $i \in \{0, A, B, AB\}$: resistance profile an infection ($i = 0$, drug-susceptible; $i = A$, resistance to Drug A; $i = B$, resistance to Drug B; and $i = AB$, resistance to both Drug A and Drug B);
- $s \in \{0,1\}$: symptom status ($i = 0$, asymptomatic, and $i = 1$, symptomatic);
- t: epidemic time;
- $N(t)$: population size at time t;
- $S(t)$: number of susceptibles at time t;
- $I_{(i,s)}(t)$: number of infected cases with resistance profile i and symptom status s at time t;
- $W_{(i,s)}(t)$: number of diagnosed cases at time t waiting to receive the first-line therapy;
- $W'_{(i,s)}(t)$: number of diagnosed cases at time t waiting to receive the second-line therapy.

The state of the gonorrhea epidemic at any given time t can be identified by a discrete-time Markov chain $\{(S(t), I_{(i,s)}(t), W_{(i,s)}(t), W'_{(i,s)}(t), i \in \{0, A, B, AB\}, s \in \{0,1\}: t = 0, \Delta t, 2\Delta t, 3\Delta t, \ldots\}$, where Δt is the time-step of the simulation (e.g. $\Delta t = 1$ day). To generate epidemic trajectories for this model, we use Monte Carlo simulation to sample from this Markov chain using the following approach. Consider a particular compartment Z in which members depart due to J events each of which is occurring at the rate $\mu_j, j \in \{1,2, \ldots, J\}$. For example, members of Susceptible compartment may leave due to 1) infection with the susceptible strain, 2) infection with Drug-A resistant strain, 3) infection with Drug-B resistant strain, or 4) infection with a strain resistant to both drugs (i.e. $J = 4$) (see Fig. S1). If the number of individuals in compartment Z at time t is $Z(t)$, then the number of individuals that leave this compartment due to events $j \in \{1,2, \ldots, J\}$ follows a multinomial distribution with total counts of $Z(t)$ and probabilities $(p_0, p_1, p_2, \ldots p_J)$, where $p_0 = 1 - e^{\sum_{j=1}^{J} \mu_j \Delta t}$ is the probability of not leaving the compartment Z during $[t, t + \Delta t]$, and $p_j = \frac{\mu_j}{\sum_{k=1}^{J} \mu_k} e^{\sum_{k=1}^{J} \mu_k \Delta t}$ is the probability of leaving the compartment Z during $[t, t + \Delta t]$ due to the event $j \in \{1,2, \ldots, J\}$.

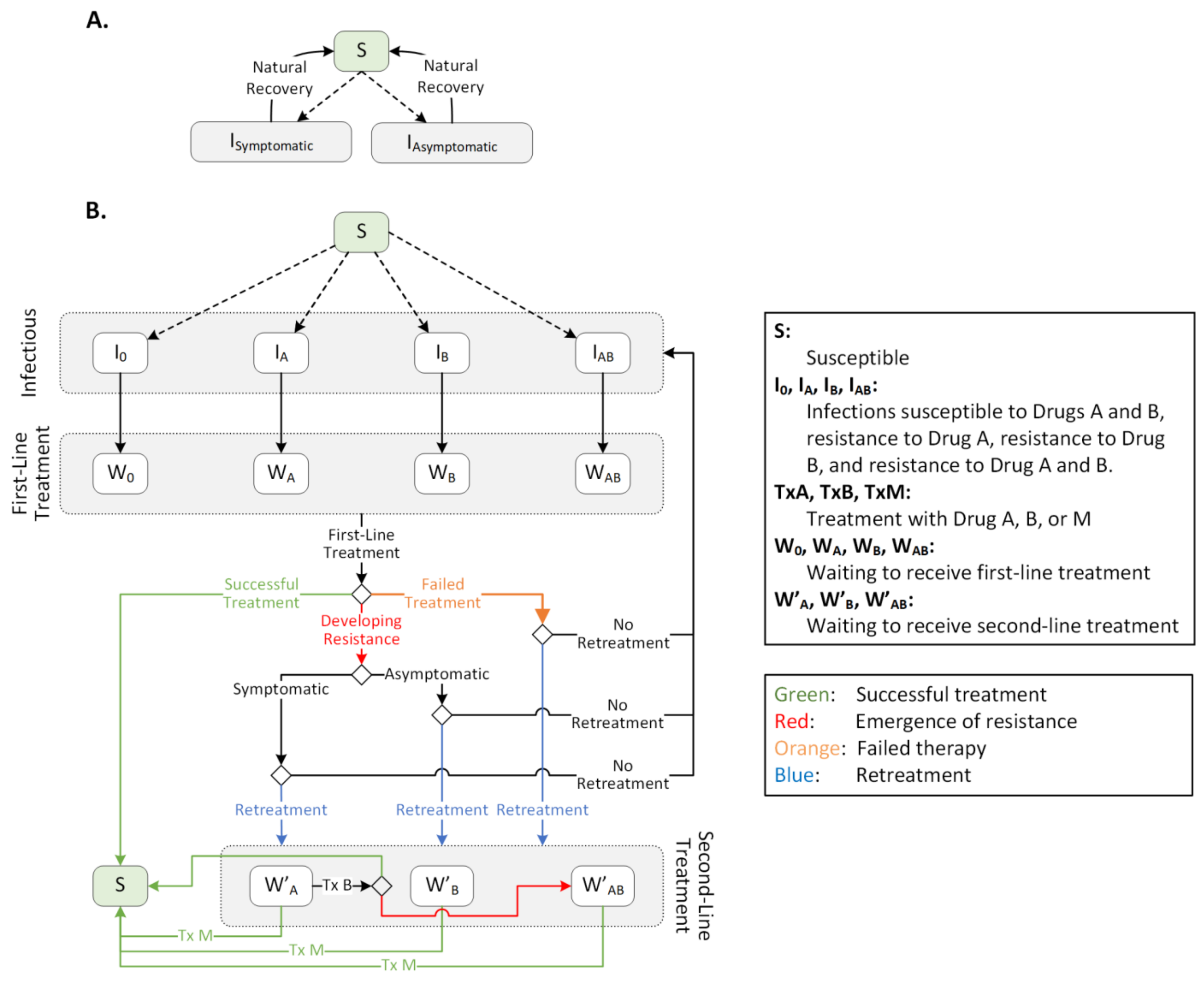

Fig. S1: Expanded model of gonorrhea transmission among the MSM population in the United States.

To identify the new epidemic state at the next time step, we first sample from the multinomial distributions associated to each compartment and then use these realizations to calculate the new epidemic state given the current epidemic state. The events that drive the epidemic are represented by arrows in Fig. S1. For example, the number of susceptibles at time $t + \Delta t$ can be calculated as:

$S(t + \Delta t) = S(t)$
$-$ new infections susceptible to Drugs A and B
$-$ new infections resistant to Drug A
$-$ new infections resistant to Drug B
$-$ new infections resistant to Drug A and Drug B
$+$ new members after natural recovery from infection
$+$ new members after successful treatment
$+$ new population members .

S1.3 Calculating the rate of infection

We calculate the daily rate of infection with resistance profile $i \in \{0, A, B, AB\}$ at time t as:

$$\mathcal{F}_i(t) = \beta_i(t) \sum_{s \in \{0,1\}} \frac{I_{(i,s)}(t) + W_{(i,s)}(t) + W'_{(i,s)}(t)}{N(t)}, \tag{1}$$

where $\beta_i(t)$ is the transmission parameter for resistance profile $i \in \{0, A, B, AB\}$. We let $\beta_0(t) = \beta$ and $\beta_i(t) = \gamma_i(t)\beta$ for $i \in \{A, B, AB\}$, where $0 \le \gamma_i(t) \le 1$ represents the fitness cost associated with the resistance profile $i \in \{A, B, AB\}$. To allow fitness cost to decrease over time, we let the relative transmissibility of the resistance profile $i \in \{A, B, AB\}$ increase over time according to:

$$\gamma_i(t) = b_{i,min} + \frac{1 - b_{i,min}}{1 + e^{-b_i(t - t_{i,0})}}. \tag{2}$$

Here, $b_{i,min} \ge 0$, $b_i \ge 0$, and $t_{i,0} \ge 0$,. Fig. S2 displays how $\gamma(t)$ changes over time and how the parameters of this function (i.e. $b_{i,min}$, b_i, and $t_{i,0}$) impact this behavior. These parameters are determined through the calibration procedure described below.

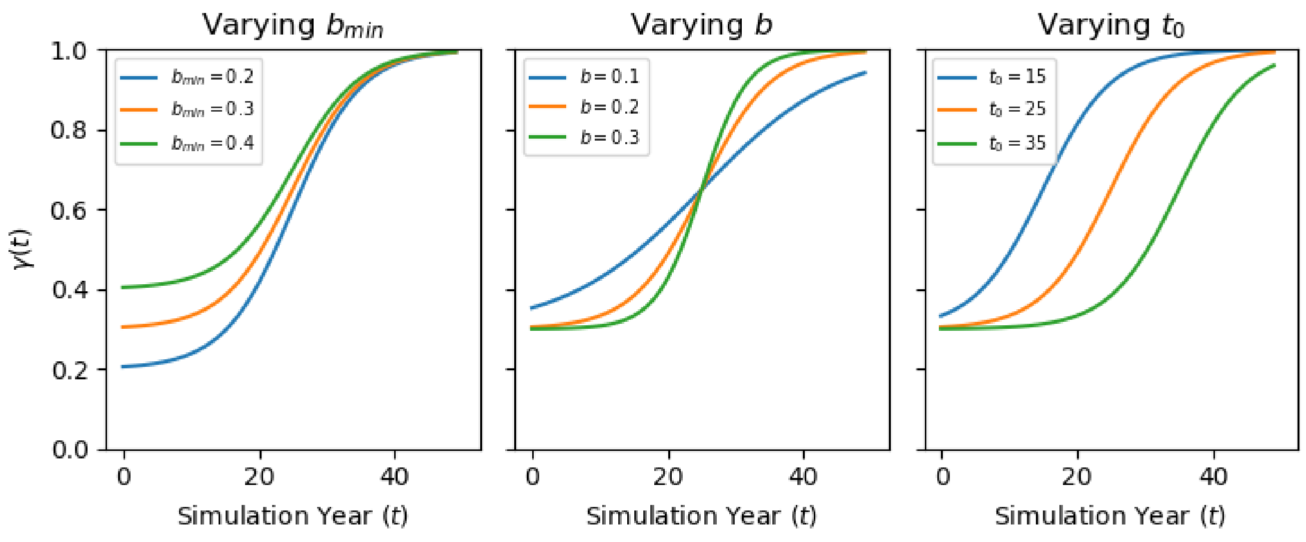

Fig. S2: Behavior of function $\gamma(t)$ (defined in Eq. (2)) over time. In these figures, the non-varying parameters are set at the default values $(b_{min}, b, t_0) = (0.3, 0.2, 25)$.

S2 Sampling error in estimating the resistance prevalence

The decision about which antibiotic to include in the first-line treatment recommendation is based on estimates of resistance prevalence obtained from surveillance systems, such as GISP [3], by evaluating a limited number of gonorrhoeae isolates for drug susceptibility. Hence, the estimates of resistance prevalence are affected by sampling error. To account for this sampling error when evaluating the policies of Table 1 using our simulation model, we use the following approach. Let y_t be the proportion of gonorrhea cases in the simulation year t that are resistant. Since not all cases are tested for drug-susceptibility, we assumed that y_t can be observed with some noise:

$$\hat{y}_t = y_t + \epsilon_t.$$

Here we assume that ϵ_t follow a normal distribution with mean 0 and standard deviation $\sqrt{y_t(1-y_t)/N}$, where N is the number of gonorrhea cases tested for drug-susceptibility. Fig. 2D-F displays the estimated proportion of cases resistant to Drugs A, B or both when $N = 5{,}000$ of annual gonorrhea cases are tested for drug resistance during each simulation. This assumption is informed by how many *N. gonorrhoeae* isolates are collected and tested through GISP in 2014 (5,093 isolates) [3].

S3 Model calibration

The model is calibrated against estimates of gonorrhea prevalence (2.0% [1.2%, 2.8%] [4,5]), the annual gonorrhea rate in 2017 (5,241.8 cases per 100,000 MSM [6]), and the proportion of gonorrhea cases with symptoms (67.9% [64.4-71.4%] [7]). To approximate the likelihood of a simulation trajectory given these observations, we chose a pseudolikelihood function consisting of three components:

S3.1 Component 1: Likelihood of gonorrhea prevalence

We assume that the 2.0% [1.2%, 2.8%] [4,5] prevalence estimate is obtained by confirming gonorrhea in $\hat{s}$ individuals out of a total of $\hat{S}$ individuals evaluated for gonorrhea (hence, $\hat{s}/\hat{S}=0.02$ and $\hat{S}$). To calculate the likelihood of observing this outcome in year t if a given simulated trajectory represents the reality, we assumed that $\hat{s}$ follows a binomial distribution with $\hat{S}$ trials and success probability τ_t, where τ_t is the prevalence of gonorrhea in year t of the simulation:

$$L_1 = \sum_{t=1}^{10} \binom{\hat{S}}{\hat{s}} \tau_t^{\hat{s}} (1-\tau_t)^{\hat{S}-\hat{s}}.$$

Here $\hat{S}$ can be approximated by noting that the half-length of the confidence interval for the estimated prevalence is:

$$HL = z_{\alpha/2}\sqrt{\frac{\mu(1-\mu)}{\hat{S}}},$$

where $\mu = \hat{s}/\hat{S}$ and $z_{\alpha/2}$ is the upper $\alpha/2$ critical point for the standard normal distribution. By using $HL = \frac{0.028-0.012}{2} = 0.08$, $\alpha = 0.05$, and $\hat{s}/\hat{S}=0.02$ in the above equation, we estimate $\hat{S}$ at 1176.

S3.2 Component 2: Likelihood of annual rate of reported gonorrhea cases

No confidence interval was reported for the estimated 5,241.8 cases of gonorrhea per 100,000 MSM in 2017 [6]. We assume that this estimate was with 20% error which is equivalent to having a reported confidence interval of [4193.4 - 6290.2]. We assume that the estimate of 5,241.8 cases of gonorrhea per 100,000 MSM in 2017 [6] is calculated as $\hat{k}/\hat{K} \times 100{,}000$, where $\hat{k}$ is the number of gonorrhea cases observed in a sample MSM population of size $\hat{K}$. To calculate the likelihood of observing this outcome in year t if a given simulated trajectory represents the reality, we assumed that $\hat{k}$ follows a binomial distribution with $\hat{K}$ trials and success probability ρ_t, where ρ_t is the proportion of the simulated population year t that got diagnosed with gonorrhea:

$$L_2 = \sum_{t=1}^{10} \binom{\hat{K}}{\hat{k}} \rho_t^{\hat{k}} (1-\rho_t)^{\hat{K}-\hat{k}}.$$

Here $\hat{K}$ can be approximated by noting that the half-length of the confidence interval for the estimated annual rate of reported gonorrhea cases is:

$$HL = 100{,}000 \times z_{\alpha/2} \sqrt{\frac{\mu(1-\mu)}{\hat{K}}},$$

where $\mu = \hat{k}/\hat{K}$ and $z_{\alpha/2}$ is the upper $\alpha/2$ critical point for the standard normal distribution. By using $HL = \frac{6290.2-4193.4}{2} = 1048.4$, $\alpha = 0.05$, and $\hat{k}/\hat{K} = 0.05242$ in the above equation, we estimate $\hat{K}$ at 1736.

S3.3 Component 3: Likelihood of proportion of gonorrhea cases that are symptomatic

The estimate for the proportion of gonorrhea cases with symptoms (67.9% [64.4-71.4%] [7]) is obtained from a study where $\hat{r} = 466$ of $\hat{R} = 686$ gonorrhea cases presented symptoms. To calculate the likelihood of observing this outcome in year t if a given simulated trajectory represents the reality, we assumed that $\hat{r}$ follows a binomial distribution with $\hat{R}$ trials and success probability y_t, where y_t is the proportion of gonorrhea cases in year t of the simulation that are symptomatic:

$$L_3 = \sum_{t=1}^{10} \binom{\hat{R}}{\hat{r}} y_t^{\hat{r}} (1-y_t)^{\hat{R}-\hat{r}}.$$

S3.4 Total pseudolikelihood

To summarize, we calculate the natural logarithm of the likelihood of observations given a simulated trajectory as:

$$\ln L = \ln L_1 + \ln L_2 + \ln L_3.$$

To improve the efficiency of the calibration procedure, we terminate the simulation of a trajectory once any of the following conditions is met:

1. Gonorrhea prevalence falls out of the range [0.5%, 6%].
2. Annual rate of reported gonorrhea cases falls out of the range [1,000, 9,000],
3. Percentage of individuals starting treatment who are symptomatic falls below 50%.

Also, to make sure that resistance to Drugs A and B emerges during the simulation horizon (50 years), we eliminate trajectories where the prevalence of resistance to Drug A never reached 5%. We also note that in calculating the

Table S1: Prior distributions and posterior intervals of model parameters

Parameter	Prior Distribution (All Uniform)	95% Posterior Interval	Sources to Inform Prior Distribution
Transmission parameter (β)	[0, 10]	(1.92, 6.41)	Assumption
Duration of infection (without treatment) (months)	[1, 60]	(5.1, 58.4)	[8]
Time until screened for infection (years)	[0.5, 5.0]	(0.6, 2.0)	[8,9]
Time until seeking treatment for a symptomatic infection (days)	[1, 14]	(1.4, 13.7)	[7,8,10]
Time until retreatment (days)	[1, 14]	(1.7, 13.6)	[7,8]
Probability that an infection will be symptomatic	[10%, 90%]	(38.1%, 67.9%)	[8,9,11]
Probability of retreatment after treatment failure with symptomatic infection	[80%, 100%]	(81.7%, 98.9%)	[9]
Probability of developing resistance while receiving Drug A	$10^{[-6, -4]}$	$10^{(-5.98, -4.02)}$	[9]
Probability of developing resistance while receiving Drug B	$10^{[-6, -4]}$	$10^{(-5.95, -4.02)}$	[9]
Relative transmissibility of the strain resistant to Drug A ($\gamma_A(t)$)			
$b_{A,min}$	[0, 1]	(0.06, 0.98)	
b_A	[0, 0.2]	(0.006, 0.182)	
$t_{A,0}$	[0, 30]	(1.0, 29.3)	
Relative transmissibility of the strain resistant to Drug B or both drugs ($\gamma_B(t)$ and $\gamma_{AB}(t)$)			
$b_{B,min}$ and $b_{AB,min}$	[0, 1]	(0.05, 0.97)	
b_B and b_{AB}	[0, 0.2]	(0.013, 0.190)	
$t_{B,0}$ and $t_{AB,0}$	[0, 40]	(1.3, 37.8)	
Initial gonorrhea prevalence	[1%, 5%]	(1.3%, 4.3%)	[4,5]
Initial proportion of gonococcal infections that are symptomatic	[0%, 50%]	(1.2%, 47.2%)	Assumption
Initial proportion of gonococcal infections resistant to Drug A	[0%, 4%]	(0.1%, 3.9%)	[3,12]
Initial proportion of gonococcal infections resistant to Drug B	[0%, 4%]	(0.2%, 3.9%)	[3,12]

likelihood functions above, we only had data for year 1 of the simulation but we assumed that the incidence and prevalence of gonorrhea and the percentage of cases who are symptomatic among the U.S. MSM population are expected to be relatively stable around the 2017 estimates over the next 10 years. Therefore, each likelihood component described above is assumed to be the sum of 10 likelihoods.

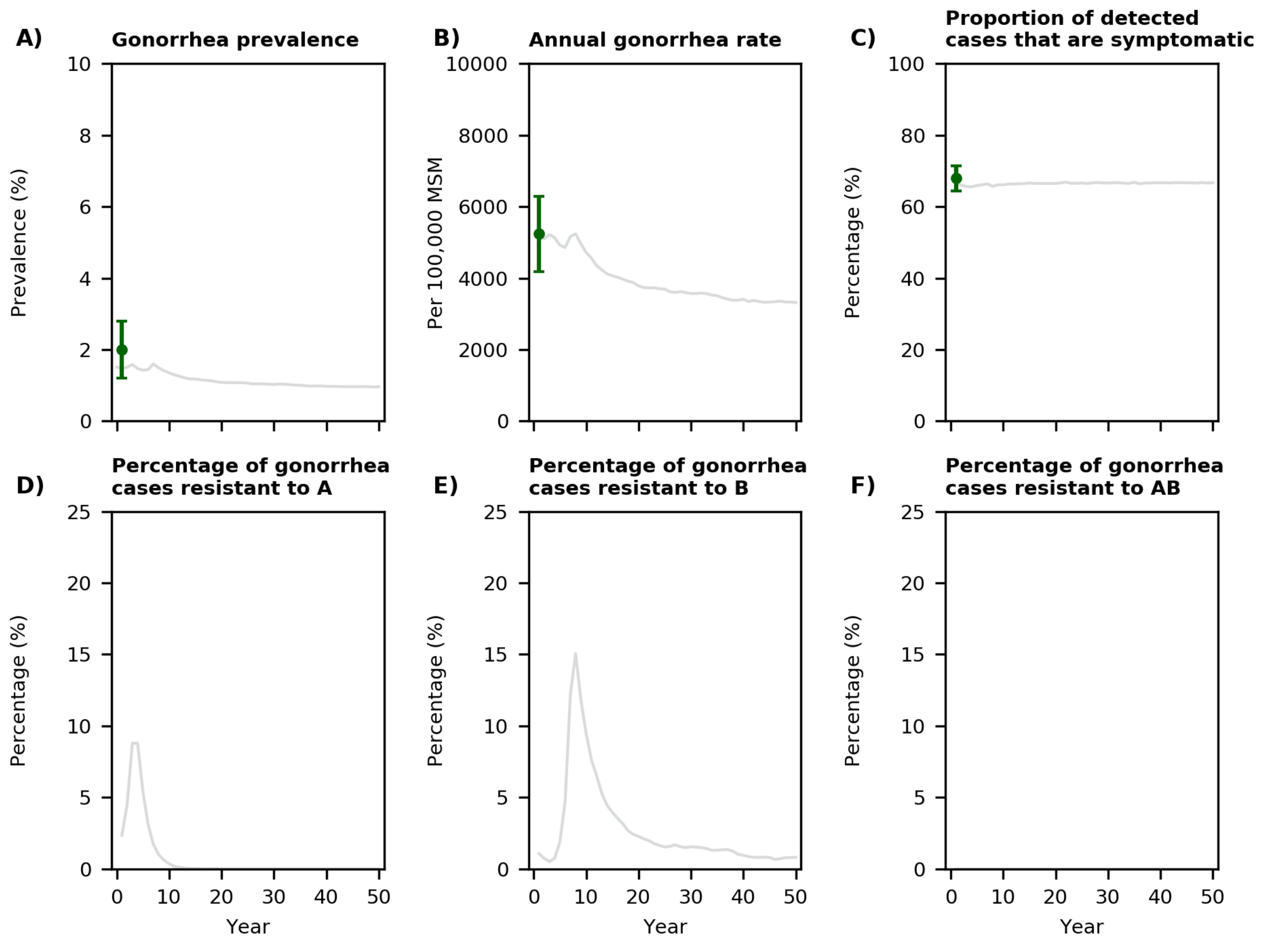

Fig. S3: Displaying an illustrative simulated trajectory from the calibrated model. The green dots in panels A-C represent the data or estimates the model is calibrated against: gonorrhea prevalence (2.0% [1.2%, 2.8%] [4,5] of MSM), the estimated rate of gonorrhea cases in 2017 (5,241.8 cases per 100,000 MSM [6]), and the proportion of gonorrhea cases among MSM that are symptomatic (67.9% [64.4-71.4%] [7]). In these simulated trajectories, the first-line treatment is changed when more than 5% of the annual gonorrhea cases are resistant to the first-line drug.

S3.5 Projections and estimating posterior distributions

To build a set of trajectories to evaluate the performance of strategies in Table 1, we used a sampling / importance sampling algorithm to approximate the posterior distributions of model parameters [13,14]. We first simulate $N_0 = 100{,}000$ epidemic trajectories, each of which uses parameter values that are randomly drawn from the prior probability distribution of epidemic parameters listed in Table S1. These prior distributions are mainly informed by estimates extracted from existing scientific literature. When such estimates are not available, we identified prior distributions by experimenting with the model ("hand-fitting") to ensure the model can produce simulated trajectories that are consistent with past observations.

Let $\ln L_i$ be the total pseudolikelihood for the simulation trajectory $i \in \{1,2,..N_0\}$. We calculated the likelihood weight of this trajectory as:

$$w_i = \frac{e^{\ln L_i}}{\sum_{j=1}^{N_0} e^{\ln L_j}}.$$

After calculating w_i for each simulated trajectory, we draw 500 trajectories, with replacement and based on likelihood weights w_i. We used the parameter values associated with these 500 trajectories to calculate the mean and 95% posterior intervals of model parameters (Table S1). Fig. S3 displays an illustrative simulation run from the calibrated model. When comparing the performance of different strategies, we use common random seeds to simulate trajectories so that a particular simulated trajectory remains the same under each strategy until the condition to switch treatment recommendations is reached.

S4 Identifying 'Threshold- Trend' strategies

Here we propose an algorithm to identify parameters of 'Threshold-Trend' strategies, τ and θ (see Table 1) that result in a superior performance compared to the 'Threshold-Annual' strategy.

Let $q(\tau,\theta,\xi)$ and $v(\tau,\theta,\xi)$ denote, respectively, the change in gonorrhea cases and in the effective lifespan of Drugs A and B under the 'Threshold-Trend' strategy with parameter (τ,θ) compared to the 'Threshold-Annual' strategy with $\tau = 5\%$, for a given simulated trajectory. Both $q(\tau,\theta,\xi)$ and $v(\tau,\theta,\xi)$ are stochastic functions and depend on a random variable ξ which represents both parameter uncertainty and random events that occur during a simulated trajectory. As discussed in the main text, minimizing $\mathrm{E}_\xi[q(\tau,\theta,\xi)]$ (i.e. averting more cases) may lead to decreasing $\mathrm{E}_\xi[v(\tau,\theta,\xi)]$ (i.e. lowering effective lifespan of Drugs A and B), and vice versa. To construct a single objective function that could be optimized, we use the net monetary benefit framework [15] and defined our objective function as $\mathrm{E}_\xi[\omega q(\tau,\theta,\xi) - v(\tau,\theta,\xi)]$, where ω represents the decision maker's willingness to increase the consumption of Drug M by one dose to avert an additional gonorrhea case over the next 50 years.

The slope of the curve at the origin of Fig. S4, which is 5.5, could be an estimate for ω if a decision maker chooses to follow the 'Threshold-Annual' strategy with 5% switch threshold. Higher switch thresholds correspond to lower ω (moving to the lower left corner of Fig. S4) and lower switch thresholds correspond to higher ω (moving toward the upper right corner of Fig. S4). We use $\tau(\omega)$ and $\theta(\omega)$ to make it explicit that these thresholds are functions of ω. Assuming that ω takes value over $[\omega_L, \omega_U]$, our goal is to characterize functions $\tau(\omega)$ and $\theta(\omega)$ that minimizes:

$$\int_{\omega_L}^{\omega_H} \mathrm{E}_\xi[\omega q(\tau(\omega),\theta(\omega),\xi) - v(\tau(\omega),\theta(\omega),\xi)]d\omega.$$

Assuming that $\tau(\omega) = \tau_0 e^{\tau_1 \omega}$ with $\tau_0 \geq 0$, and $\tau_1 \leq 0$, and $\theta(\omega) = \theta_0 \tau(\omega)$ with $0 \leq \theta_0 \leq 1$, we solve the following optimization problem to characterize $\tau(\omega)$ and $\theta(\omega)$:

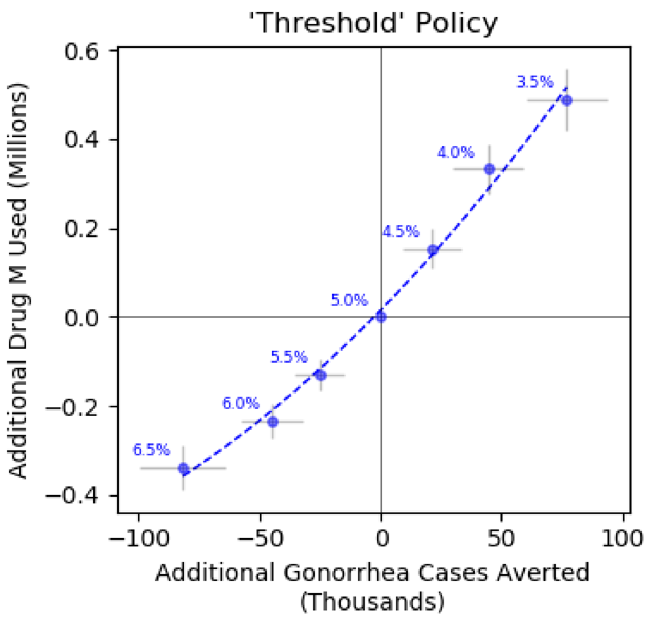

Fig. S4: The impact of changing the switch threshold of the 'Threshold' policy (Table 1) on the consumption of Drug M and the cases of gonorrhea averted over the 50 years of simulation.

$$\min_{\tau_0,\tau_1,\theta_0} \int_{\omega_L}^{\omega_H} \mathrm{E}_\xi[\omega q(\tau(\omega),\theta(\omega),\xi) - v(\tau(\omega),\theta(\omega),\xi)]d\omega \quad (1)$$

$$\text{Subject To: } \tau(\omega) = \tau_0 e^{\tau_1 \omega},$$

$$\theta(\omega) = \theta_0 \tau(\omega),$$

$$\tau_0 \geq 0,$$

$$\tau_1 \leq 0,$$

$$0 \leq \theta_0 \leq 1.$$

We solve the optimization problem (1) using a stochastic approximation algorithm described below.

S4.1 Stochastic approximation algorithms

The goal of stochastic approximation (SA) algorithms [16,17] is to find the minimizer of a function

$$f(x) = \mathrm{E}_\xi[F(x,\xi)], \quad (2)$$

which is the expected value of a stochastic function $F(\cdot)$ that depends on a random variable ξ. An example of $F(\cdot)$ could be the total number of gonorrhea cases during the next 20 years. It is a stochastic function since its value depends on many random events (represented by ξ) that may occur during each period. A simple version of SA algorithms generates the sequence of iterates:

$$x_{n+1} = x_n - p_n \frac{y_n}{\|y_n\|}, \quad (3)$$

where y_n is an unbiased estimate of the derivative of f at x (i.e. $\nabla f(x_n)$), $\|y\|$ is the Euclidean norm of the vector y, and p_n is a sequence of positive step sizes with the properties that $p_n \to 0$ and $\sum_n p_n = \infty$ (e.g., $p_n = \frac{a_0 b}{n+b}$, with $a_0 \geq 0$ and $b \geq 1$).

Table S2: Stochastic Approximation algorithm to find the minimum (x^*) of a function $f(x) = \mathrm{E}_\xi[F(x, \xi)]$

1. Choose the number of iterations N.
2. Choose an initial value for x (denoted by x_0)
3. Choose step size rule: $p_n = \frac{a_0 b}{n+b}$, with $a_0 \geq 0$ and $b \geq 1$.
4. Choose Step size rule for derivatives: $\epsilon_n = c_0 \sqrt[-4]{n}$, with $c_0 \geq 0$.
5. For $n = 0$ to N:
   a. Set f_n to a realization of $F(\cdot)$ at x_n (i.e. $F(x_n, \xi)$).
   b. Estimate the derivative of f at x_n according to Eq. (4).
   c. Set $x_{n+1} \leftarrow x_n - p_n \frac{y_n}{\|y_n\|}$.
6. Return $x^* = \sum_{n=N-M}^{N} x_n / M$ and $f^* = \sum_{n=N-M}^{N} f_n / M$, where M is the number of last iterations to use to calculate x^* and f^* (e.g., $M = 0.2N$).

The derivative estimate $y_n = (y_n^1, y_n^2, \dots, y_n^K)$ can be obtained by:

$$y_n^i = \frac{F\left(x_n^i + \epsilon_n e_i, \xi\right) - F(x_n^i - \epsilon_n e_i, \xi)}{2\epsilon_n}, i = 1,2, \dots, K, \quad (4)$$

where e_i is a vector with 1 in the ith element and 0 elsewhere and ϵ_n is a sequence of positive step sizes with the property that $\epsilon_n \to 0$. ϵ_n is selected such that it approaches 0 at a slower rate than p_n (e.g., $\epsilon_n = c_0 \sqrt[-4]{n}$, with $c_0 \geq 0$). One way to reduce the noise in estimating the derivatives is to use the same stream of random numbers in generating the realizations $F\left(x_n^i + \epsilon_n e_i, \xi\right)$ and $F(x_n^i - \epsilon_n e_i, \xi)$ when calculating y_n^i's. The pseudo-code of this algorithm is provided in Table S2.

S4.2 Optimization settings

To find $(\tau_0, \tau_1, \theta_0)$ that optimizes problem (1), we applied the stochastic approximation algorithm in Table S2 with the following settings:

$$F(\tau_0, \tau_1, \theta_0; \xi) = Q(\tau_0, \tau_1, \theta_0; \xi) + \Upsilon([\text{neg}(\tau_0)]^2 + [\text{neg}(-\tau_1)]^2 + [\text{neg}(\theta_0)]^2 + [\text{neg}(1 - \theta_0)]^2), \quad (5)$$

where $\text{neg}(x) = x$ if $x < 0$, and $\text{neg}(x) = 0$ if $x \geq 0$, Υ is the penalty factor to penalize a $(\tau_0, \tau_1, \theta_0)$ that violates the feasibility constraints of the optimization problem (1), and

$$Q(\tau_0, \tau_1, \theta_0; \xi) = \frac{1}{3} \sum_{i=1}^{3} [\omega_i q(\tau(\omega_i), \theta(\omega_i); \xi) - v(\tau(\omega_i), \theta(\omega_i); \xi)] \quad (6)$$

is an approximation for the objective function (1).

In Eq. (6), we set $\omega_1, \omega_2, \omega_3$ to the slope of the curve in Fig. S4 at the smallest threshold, the 5% threshold, and the largest threshold, respectively (3.5, 5.5, 7.5). For the results presented here, we applied stochastic approximation algorithm in Table S2 with $N = 1000$, $M = 200$ and selected $\Upsilon = 10^6$ for the penalty factor in Eq. (5). To optimize a policy, we ran the algorithm with $a_0 \in \{0.05, 0.1\}$, $b \in \{10, 25, 50\}$ and $c_0 \in \{0.05, 0.1\}$ and selected $(\tau_0, \tau_1, \theta_0)$ that resulted in the highest f^* across all combinations of a_0, b, and c_0. Fig. S5 displays an example of 'Threshold + Trend' policy.

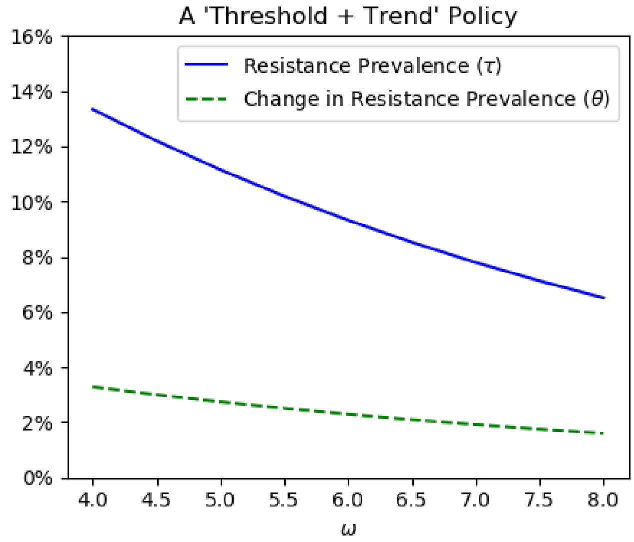

Fig. S5: A 'Threshold-Trend' Policy uses two thresholds to inform switching: resistance prevalence (**τ**) and percentage point change in the resistance prevalanece (**θ**). Here **ω** represents years of the effective lifespan of Drugs A and B that a decision maker is willing to sacrifice to avert an additional gonorrhea case per 100,000 MSM population per year.

S5 Sensitivity Analysis

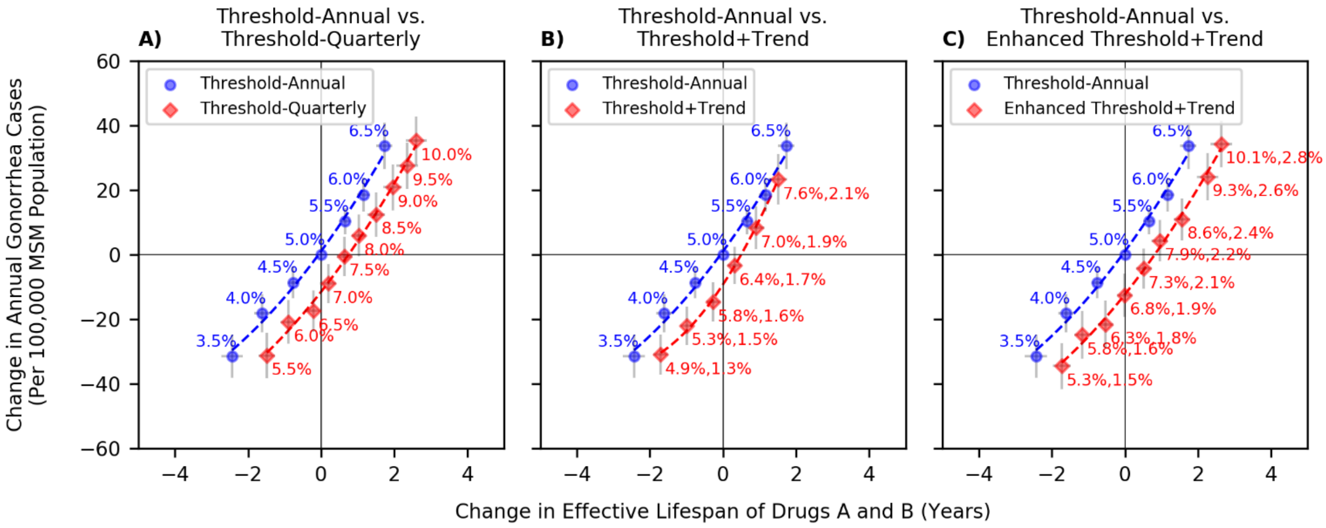

Fig. S6: Comparing the performance of policies in Table 1 with respect to the current policy over a 25-year simulation window. The origins in these figures reflect the current policy that recommends switching the antibiotic used for empiric treatment once the estimated resistance prevalence exceeds 5% [18,19]. The numbers on the curves of 'Threshold-Annual' and 'Threshold-Quarterly' strategies represent the threshold of resistance prevalence to switch the first-line therapy of gonorrhea, and the two numbers on the curves of 'Threshold+Trend' and 'Enhanced Threshold+Trend' strategies represent the two thresholds used to inform switching: resistance prevalence (first %) and percentage point change in the resistance prevalence (second %).

References

1. Purcell DW, Johnson CH, Lansky A, Prejean J, Stein R, Denning P, et al. Estimating the population size of men who have sex with men in the United States to obtain HIV and syphilis rates. Open AIDS J. 2012;6:98-107. Epub 2012/10/11. doi: 10.2174/1874613601206010098. PubMed PMID: 23049658; PubMed Central PMCID: PMCPMC3462414.
2. U.S. Census Bureau CPS, Annual Social and Economic Supplement,. Age and Sex Composition in the United States: 2016 2016. Available from: https://www.census.gov/data/tables/2016/demo/age-and-sex/2016-age-sex-composition.html.
3. Kirkcaldy RD, Harvey A, Papp JR, Del Rio C, Soge OO, Holmes KK, et al. *Neisseria gonorrhoeae* Antimicrobial Susceptibility Surveillance - The Gonococcal Isolate Surveillance Project, 27 Sites, United States, 2014. MMWR Surveill Summ. 2016;65(7):1-19. Epub 2016/07/16. doi: 10.15585/mmwr.ss6507a1. PubMed PMID: 27414503.
4. Fingerhuth SM, Bonhoeffer S, Low N, Althaus CL. Antibiotic-Resistant Neisseria gonorrhoeae Spread Faster with More Treatment, Not More Sexual Partners. PLoS Pathog. 2016;12(5):e1005611. Epub 2016/05/20. doi: 10.1371/journal.ppat.1005611. PubMed PMID: 27196299; PubMed Central PMCID: PMCPMC4872991.
5. Grov C, Cain D, Rendina HJ, Ventuneac A, Parsons JT. Characteristics Associated With Urethral and Rectal Gonorrhea and Chlamydia Diagnoses in a US National Sample of Gay and Bisexual Men: Results From the One Thousand Strong Panel. Sex Transm Dis. 2016;43(3):165-71. Epub 2016/02/10. doi: 10.1097/OLQ.0000000000000410. PubMed PMID: 26859803; PubMed Central PMCID: PMCPMC4748382.
6. Centers for Disease Control and Prevention. Sexually Transmitted Disease Surveillance 2017. Atlanta, U.S.: U.S. Department of Health and Human Services, 2018.
7. Newman LM, Dowell D, Bernstein K, Donnelly J, Martins S, Stenger M, et al. A tale of two gonorrhea epidemics: results from the STD surveillance network. Public Health Rep. 2012;127(3):282-92. Epub 2012/05/02. doi: 10.1177/003335491212700308. PubMed PMID: 22547859; PubMed Central PMCID: PMCPMC3314072.
8. Whittles LK, White PJ, Didelot X. Estimating the fitness cost and benefit of cefixime resistance in Neisseria gonorrhoeae to inform prescription policy: A modelling study. PLoS Med. 2017;14(10):e1002416. Epub 2017/11/01. doi: 10.1371/journal.pmed.1002416. PubMed PMID: 29088226; PubMed Central PMCID: PMCPMC5663337.
9. Tuite AR, Gift TL, Chesson HW, Hsu K, Salomon JA, Grad YH. Impact of Rapid Susceptibility Testing and Antibiotic Selection Strategy on the Emergence and Spread of Antibiotic Resistance in Gonorrhea. J Infect Dis. 2017;216(9):1141-9. Epub 2017/10/03. doi: 10.1093/infdis/jix450. PubMed PMID: 28968710; PubMed Central PMCID: PMCPMC5853443.
10. Garnett GP, Mertz KJ, Finelli L, Levine WC, St Louis ME. The transmission dynamics of gonorrhoea: modelling the reported behaviour of infected patients from Newark, New Jersey. Philos Trans R Soc Lond B Biol Sci. 1999;354(1384):787-97. Epub 1999/06/12. doi: 10.1098/rstb.1999.0431. PubMed PMID: 10365404; PubMed Central PMCID: PMCPMC1692556.
11. Hui BB, Whiley DM, Donovan B, Law MG, Regan DG, Investigators GS. Identifying factors that lead to the persistence of imported gonorrhoeae strains: a modelling study. Sex Transm Infect. 2017;93(3):221-5. Epub 2017/04/23. doi: 10.1136/sextrans-2016-052738. PubMed PMID: 28432206.
12. Kirkcaldy RD, Hook EW, 3rd, Soge OO, del Rio C, Kubin G, Zenilman JM, et al. Trends in Neisseria gonorrhoeae Susceptibility to Cephalosporins in the United States, 2006-2014. JAMA. 2015;314(17):1869-71. Epub 2015/11/04. doi: 10.1001/jama.2015.10347. PubMed PMID: 26529166; PubMed Central PMCID: PMCPMC4788090.
13. Menzies NA, Soeteman DI, Pandya A, Kim JJ. Bayesian Methods for Calibrating Health Policy Models: A Tutorial. Pharmacoeconomics. 2017. doi: 10.1007/s40273-017-0494-4. PubMed PMID: 28247184.
14. Poole D, Raftery AE. Inference for deterministic simulation models: The Bayesian melding approach. J Am Stat Assoc. 2000;95(452):1244-55. doi: Doi 10.2307/2669764. PubMed PMID: WOS:000165470300026.

15. Stinnett AA, Mullahy J. Net health benefits: a new framework for the analysis of uncertainty in cost-effectiveness analysis. Med Decis Making. 1998;18(2 Suppl):S68-80. Epub 1998/05/05. doi: 10.1177/0272989X98018002S09. PubMed PMID: 9566468.
16. Chau M, Fu MC. An Overview of Stochastic Approximation. Int Ser Oper Res Man. 2015;216:149-78. doi: 10.1007/978-1-4939-1384-8_6. PubMed PMID: WOS:000357825100007.
17. Fu MC. Optimization for simulation: Theory vs. practice. Informs J Comput. 2002;14(3):192-215. doi: DOI 10.1287/ijoc.14.3.192.113. PubMed PMID: WOS:000177537400002.
18. Bolan GA, Sparling PF, Wasserheit JN. The emerging threat of untreatable gonococcal infection. N Engl J Med. 2012;366(6):485-7. Epub 2012/02/10. doi: 10.1056/NEJMp1112456. PubMed PMID: 22316442.
19. World Health Organization. Global action plan to control the spread and impact of antimicrobial resistance in Neisseria gonorrhoeae. Geneva: 2012.
