# Supplementary material for: Adaptive guidelines for the treatment of gonorrhea to increase the effective life span of antibiotics among men who have sex with men in the United States: A mathematical modeling study
Source: PLoS Med. 2020 Apr 3;17(4):e1003077. doi: 10.1371/journal.pmed.1003077 (PMC7122693; doi:10.1371/journal.pmed.1003077)
Supplement: Attachment — Submitted filename: Response Letter.pdf [file pmed.1003077.s002.pdf]

Yale SCHOOL OF PUBLIC HEALTH
Department of Health Policy and Management

Reza Yaesoubi, Ph.D.
Assistant Professor
Yale School of Public Health

courier
60 College Street, Room 314
New Haven, CT 06510

203 785-6982
reza.yaesoubi@yale.edu

October 22, 2019

Dear *PLOS Medicine* Editors:

We appreciate the opportunity to present a revised version of our manuscript (tracking number: PMEDICINE-D-19-02321): "Adaptive guidelines for the treatment of gonorrhea to increase the effective lifespan of antibiotics: A mathematical modeling study."

We would like to thank all reviewers for their insightful comments and specific, constructive suggestions on how we could improve both the content and presentation of our manuscript. We have been able to address each of the suggestions and concerns, and believe that the manuscript is stronger and more clearly presented as a result of these revisions. Below, we provide point-by-point responses.

Thank you for your consideration of this revised manuscript. We look forward to your evaluation and are available if questions should arise.

Sincerely,

Reza Yaesoubi,
Ted Cohen,
Katherine Hsu,
Thomas L. Gift,
Harrell Chesson,
Joshua A. Salomon,
Yonatan H. Grad

INNOVATION THROUGH COLLABORATION
100
Yale School of Public Health
1915–2015

Responses to Requests from the Editors:

(1) "In the Abstract Methods and Findings section, please include a bit more information on the population and setting (i.e., the cohort from which you derive the inputs), the specific policy changes investigated, the timeframe simulated, and the main outcome measures."

Response: We have revised the Abstract to provide more information on the population, policy changes we investigated, timeframe of simulation, and the main outcome measure.

(2) "Also in the Abstract Methods and Findings, please quantify the main results (with 95% UIs and p values where relevant)"

Response: The revised abstract now includes estimates of main outcomes.

> "…extend the effective lifespan of antibiotics by 0.83 years without increasing the number of gonorrhea cases. This is equivalent to successfully treating an additional 86.8 (95% uncertainty interval: [51.7, 121.2]) gonorrhea cases per 100,000 MSM population each year with the first-line antibiotics without worsening the burden of gonorrhea."

(3) "At this stage, we ask that you include a short, non-technical Author Summary of your research to make findings accessible to a wide audience that includes both scientists and non-scientists. The Author Summary should immediately follow the Abstract in your revised manuscript. This text is subject to editorial change and should be distinct from the scientific abstract."

Response: The Author Summary is included.

(4) "In the first paragraph of the Discussion, please summarize what was done before describing the findings."

Response: The first paragraph of the Discussion section now briefly summarizes what was done in this study.

(5) "Please present and organize the Discussion as follows: a short, clear summary of the article's findings; what the study adds to existing research and where and why the results may differ from previous research; strengths and limitations of the study; implications and next steps for research, clinical practice, and/or public policy; one-paragraph conclusion."

Response: We revised the Discussion section to conform with this structure.

Responses to Reviewer 1

“While I think this is a really nice and important piece of work, the manuscript appears a little incomplete and might benefit from some further investigation. I have a number of suggestions that the authors could consider in a revised version of the manuscript which, I believe, would considerably help strengthen the overall conclusions:”

(1) “Sensitivity analyses: My impression is that the results of the paper might heavily depend on a number of critical assumptions. For example, it is unclear to me why the authors chose a time window of 50 years. Also, increasing the number of additional antibiotics (C, D, ..., or N, K, ...) could result in quite some different results. I think some sensitivity analyses in that respect might be warranted.”

Response: The simulation window of 50 years was selected to ensure enough time for the resistance to emerge against Drug A and Drug B. In the revised manuscript, we present our main findings when simulations are run for 25 and 50 years (Fig. 3 and Fig. S6). These figures show that our conclusions are not sensitive to the simulation length.
Regarding the number of available antibiotics, we chose to include 3 antibiotics in our analysis since this is a conservative strategy that matches the antibiotics currently available (e.g., ceftriaxone and azithromycin) plus another, representing an antibiotic used to treat gonorrhea without a formal recommendation (e.g., ertapenem).

(2) “Uncertainty: The authors write that they summarize results "using the mean and 95% confidence intervals across 500 simulated trajectories". However, I did not find any reporting on confidence intervals or any assessment of uncertainty. Fig. 3 is truly excellent and contains an incredible amount of information, but one wonders how much uncertainty is expected there. Table 1 could also be extended with some key results for the resistance thresholds of 5% and 10%, for example.”

Response: Thank you for pointing out this issue. The error bars had been accidently eliminated from Fig 3. They are now displayed in this figure. We appreciate the suggestion of extending Table 1. We envisioned Table 1 as presenting the definition of various adaptive guidelines we investigated in the manuscript. To keep this Table simple, we are presenting the estimated outcomes of each strategies under different resistance thresholds in Fig 3. It is not clear to us how best to follow the Reviewer’s suggestion to include the results in Table 1. We would welcome specific suggestions on how to do so.

(3) “Methods: I found it a bit difficult to completely follow the modeling procedure. First, it is unclear why the authors chose the simulation approach described in S1.2 instead of the exact stochastic Gillespie algorithm. Did the authors opt for a fixed time step because of computational constraints? Second, it is also not clear how model calibration is performed. Apparently, the authors run 100,000 trajectories but it is not described how the posterior distributions are obtained based on the likelihood. Finally, I could not exactly follow what happens in the threshold-trend strategies described in S4. It appears the authors want to identify tau and theta, but I could not find any reporting of these values (at least not of theta) in the manuscript. Some additional details on this specific scenario and its results would be helpful.”

Response: Thank you for these suggestions.

1. The Reviewer is correct about the choice of our discrete-time simulation approach. Gillespie algorithm is computationally more expensive especially for large populations. We therefore used

a discrete-time updating algorithm as an approximation to the exact Gillespie algorithm to speed up the simulations. This algorithm is descried in §S1.2 of the Supplementary Information text.

2. We used a sampling / importance sampling algorithm to approximate the posterior distributions of model parameters. We added a new subsection in the Supplementary Information text ("S3.5 Projections and estimating posterior distributions") to describe the details of the algorithm.

3. We have included the parameter values of 'Threshold-Trend' strategy in Fig 3B-C. We have also added a new figure in the Supplementary Information text (Fig. S5) to show how the threshold for resistance prevalence (τ) and the threshold for the change in resistance prevalence (θ) changes as the function of ω which represents the decision maker's willingness to increase the consumption of Drug M by one dose to avert an additional gonorrhea case.

(4) "Minor points:"

a. "Figure 2: It would help if the panels in this figure highlighted the median of all simulations, or an illustrative or typical simulation run."

Response: We have included a figure in the Supplementary Information text (Fig S3) to display an illustrative simulation run.

b. "Supplementary Information, p. 5, line 423: "estimated" should be "estimate"."

Response: Thank you. We corrected the error.

Responses to Reviewer 2

"The manuscript represents a valuable study into the potential impacts of both switching and optimising criteria for moving between first and second line and second and last line antibiotic therapies. The study is almost entirely a simulation study. A detailed, yet realistic stochastic epidemic transmission model is established, which has a number of parameters as presented in Table 1. I have one query about the model:"

Major Comments

(1) "In the SI, in Section 1.3, t_0 is defined to be the time at which the increase in the relative infectiousness of resistance profile i should occur. How is this time determined? It's not in the parameter list. Is γ_i(t) a constant before this time?"

Response: We have revised §S1.3 of the Supplementary Text and added a new figure (Fig S2) to better describe the behavior of function $\gamma_i(t)$, which we used to capture how the fitness cost of resistance to an antibiotic changes over time. The parameters of function $\gamma_i(t)$ are determined through the calibration procedure described in §S3. The prior distributions and posterior ranges of these parameters are included in Table S1.

"Model outputs are then related to three data points - two of which are extracted from published estimates and confidence intervals. For these data points an approximate likelihood is drawn up. The analysis of these data points is claimed to be Bayesian."

(2) "The likelihood is defined, but it is unclear how exactly it is used to produce the posterior distributions. The paper says that N = 100,000 epidemic trajectories are simulated using parameters sampled from the prior distributions. Some trajectories are not considered if they don't satisfy various feasibility constraints."

a. "On the remaining trajectories, what happens? Each will have an associated likelihood value, but it is not clear how these are used to produce the posterior distributions? Is MCMC used somehow? Are only a further proportion of the trajectories retained on the basis of their likelihood? This really isn't clear and left me rather confused. It looks like only four parameters have posterior distributions that are drastically different from their prior - which makes sense given there are only three data points."

Response: We apologized for the lack of clarity around the calibration procedure. We used a sampling / importance sampling algorithm to approximate the posterior distributions of model parameters. We added a new subsection in the Supplementary Information text ("S3.5 Projections and estimating posterior distributions") to describe the details of the algorithm.

b. "I think there has been a typo in the range of the priors and posteriors for the initial gonorrhea prevalence, as they are not consistent with each other."

Response: Thank you for pointing out the error in the range of the prior and posterior for the initial gonorrhea prevalence.

c. Also, minor point, but for parameters such as the probability of drug resistance, the prior specified is uniform, but it seems that the order of magnitude is uncertain. The current uniform prior places very little prior probability on this being 10^-5 or 10^-6, which is not the intention, I'm sure. It would make more sense to place the prior on the log-scale."

Response: Thank you for this suggestion. In the revised analysis, we assumed that the probability of developing resistance while receiving Drug A or Drug B is calculated as 10^{-u}, where the prior distribution of u follows a Uniform $[4, 6]$.

(3) "Does removing trajectories that never give a prevalence of resistance of 5% bias against strategies that use a thresholding rule <5%? Are the three criteria in lines 440-442 really so infeasible over the next 50 years?"

Response: Depending on the combination of parameter values, resistance against antibiotics may never arise. Including trajectories for which resistance never occurs would underestimate the differences in the performance of different strategies because for these trajectories all strategies perform the same as decisions to revise first-line therapy are never made. We reran the calibration procedure and eliminated trajectories where the prevalence of resistance never reached 3.5% and we ended up with the same trajectories as when we used the 5% threshold to eliminate trajectories. This is because that for trajectories for which the prevalence of resistance reaches 3.5%, the prevalence of resistance continued to rise to 5% when no changes were made to the first-line treatment.

Regarding the feasibility ranges, since our model is not structured to capture the trend in the incidence and prevalence of gonorrhea among the MSM, we ran our analysis under the assumption that the incidence and prevalence of gonorrhea among this population would remain more or less stable over time. These feasibility ranges are assumed to speed up the calibration procedure by terminating the simulation of trajectories that fall outside these ranges. We have added a sentence to the Discussion to describe this assumption as one of the limitations of our analysis:

> "While data from surveillance systems indicate an upward trend in the rate of gonorrhea cases among the MSM (1), we assumed that the incidence and prevalence of gonorrhea among this population are expected to be relatively stable around the 2017 estimates (Fig. 2)."

(4) "There are many ways to calculate a confidence interval on the basis of binomial data. However, I don't think it is reasonable to be anticipating that the width of such an interval is based on the quantiles of a t-distribution. t-tests are used in cases where the variance of the data is unknown and has to be estimated separately to the mean. However, the estimate for variance of binomial data follows once the probability parameter has been estimated. Therefore, a z-statistic would be used. Can the authors justify their use of the t-distribution rather than the z? Given the likely sample sizes being estimated, this is very unlikely to make any real difference to the analyses, though does simplify the calculations. There's also a typo in the SI on line 420, it should be K hat, not S hat that is being estimated."

Response: We agree with the Reviewer that the use of z-statistics here would be more appropriate (and simplify the calculations). We revised this subsection accordingly. And thank you for pointing out the typo. We changed $\hat{S}$ to $\hat{K}$.

"Of the retained trajectories, the impact of the four considered policies are assessed, with a detailed description of how each of the policies are optimised."

(5) "The Results section discusses some improvements that could be made given certain thresholds. It would be good to state explicitly (perhaps I missed it) if these thresholds are the ones found through the optimisation process outlined in the SI?"

Response: Thank you for this suggestion. We have added this sentence to the Method section to clarify:

> "For the 'Threshold + Trend' and 'Enhanced Threshold + Trend' strategies (Table 1), the two thresholds used to inform switching (i.e. threshold for resistance prevalence and the threshold for change in the resistance prevalence) are determined using the optimization algorithm described in §S4 of the Supplementary Information text."

(6) "Have any values of ω been used previously? Also, there is no discussion of the anticipated costs of the enhanced surveillance ... are the improvements in the extension of the useful life of the antibiotics cost-efficient?"

Response: To our knowledge, estimates for ω (the trade-off between the objective of increasing effective lifespan of antibiotics and the objective of preventing gonorrhea cases) has not be reported or used previously. We agree with the Reviewer that enhancing surveillance would add to the cost of surveillance. We have discussed this in the Discussion section:

> "Enhancing surveillance systems to enable more frequent reporting and evaluation of more gonococcal isolates would increase the cost of surveillance. While the cost-effectiveness of these proposed changes needs to be studied, the analysis presented here highlights the importance of maintaining effective surveillance systems and the engagement of policy makers to turn surveillance findings into timely decisions to better control the spread of drug-resistant gonorrhea."

(7) "The final paragraph of the SI discusses ω_1, ω_2, ω_3 being drawn from the minimum, origin and maximum values from Fig. 3. I'm right in thinking these are not conventional minima and maxima, rather that they just come from the gradient at the largest and smallest values of the threshold that were considered?"

Response: The Reviewer is correct. We modified the sentence to clarify:

> "… we set $\omega_1, \omega_2, \omega_3$ to the slope of the curve in Fig. S4 at the smallest threshold, the 5% threshold, and the largest threshold, respectively (3.5, 5.5, 7.5)."

(8) "Minor point, there are some typos in formulae:"

- **a.** "should there be a '-' in Equation (6) of the SI, rather than a '+'?"

  Response: Thank you for pointing out this error. The '+' sign has changed to a '−' sign.

- **b.** "in line 484, p_n approaches zero at a slower rate than p_n? I think the first should be ε_n."

  Response: Thank you again. Corrections are made.

c. "the equation in line 356 is confusing, if j is the index on the LHS, it shouldn't be used in the summations, which should be switched to k or some other letter."

Response: Thank you. The index j in the right-hand side is replaced with k.

d. "re-use of notation β in Section 3.3 of the SI. This is also used for the transmissibility. Could a different symbol be used?"

Response: Thank you. We replaced β with y.

Responses to Reviewer 3

"This manuscript presents a transmission modelling study of innovative drug cycling strategies for the treatment of gonorrhea in the US MSM population. The ideas are intriguing and the ideas described here are important.

Unfortunately, I found it slightly too theoretical for PLOS Medicine.

There are only a few drugs used routinely around the world for treating this pathogen and their rates of acquiring resistance and their initial drop in fitness has been quantified. In a piece aimed at influencing policy, given these other findings are out there, it doesn't seem reasonable to drop back to a purely theoretical Drug A versus Drug B versus Drug N. The direction of effects of more rapid decisions or higher thresholds are reasonably intuitive (but still need pointing out). Readers of this style of article at PM will be looking for robust evidence that the strength of the effect has been accurately assessed - or if not accurately, at least as accurate as is possible with current data and approaches.

Although much of the language was clear and the charts were well designed. There were key aspects of the reporting that I couldn't appreciate even on careful reading. See comments about my ability to interpret magnitude for both the x and y axis of figure 3. I suspect that the value of this figure is higher than I was able to see, but it was quite opaque to me.

Given the call out to the UK policy in the discussion, it seemed strange to me that the authors were not influenced more by doi.org/10.1371/journal.pmed.1002416 in this journal. I don't know for sure, but I think this prior paper directly influenced the decision they mention.

Just to reiterate - there is genuine innovation here and a whole set of possible strategies not looked at before in the literature. But this presentation of the ideas did not seem to be specific enough to justify the strong statements to policy-making readers of the journal."

Detailed Comments

(1) "Line 34; The caveas in the results section seemed a little strange. Maybe in the conclusions instead?"

Response: Here, we followed PLOS Medicine's submission guideline (https://journals.plos.org/plosmedicine/s/submission-guidelines#loc-abstract), which indicates that the main limitations of the study should be discussed under "Methods and Findings" section of the Abstract. We are happy to revise if the editors and reviewers would prefer an alternative.

(2) "42; Seems unusual not to have mentioned the modelling study by Whittles et al that appeared in this journal. They quantified relative fitness of different strains using a model, which seems highly relevant here."

Response: Thank you. We have now cited the study by Whittles et al. in several places. In particular, the following sentence is added to the Discussion section:

> "We assumed that once an antibiotic treatment for gonorrhea is abandoned because of the level of resistance, it will not be reintroduced. However, alternative stewardship and diagnostic strategies (e.g. the use of sequence-based diagnostics to identify the resistance profile [19]) suggest the possibility of reintroduction of these antibiotics. For example, a recent modeling study suggests that cefixime, which had previously been removed from clinical use due to increasing levels of resistance, could be

reintroduced to treat a minority of cases, assuming that cefixime resistance incurs a fixed fitness cost [20].

(3) "99; Maybe call this threshold-annual"

Response: Thank you. The strategy 'Threshold' is renamed to 'Threshold-Annual'.

(4) "130; How much less transmissible. That seems like a key assumption that needs some discussion. Ref 15 doesn't seem to have any specific estimates of relative transmissibility"

Response: We have provided additional details about this assumption in §S1.3 of the Supplementary Information text. We also added a new figure (Fig S2 in Supplementary Information) to visualize how the relative transmissibility of a resistance profile is assumed to change over time.

(5) "133; How exactly is this done and what are the approximate ranges for initial fitness cost and eventual costs."

Response: Please see our response to the comment 4 above. The parameters that determine the behavior of function $\gamma_i(t)$, which models how relative transmissibility of resistance profile i changes over time, are estimated through the calibration procedure described in §S3 of Supplementary Information.

(6) "141; A table of key parameter assumptions, prior distributions and posterior estimates would be very helpful here"

Response: Thank you. Table S1 in the Supplementary Information text lists the prior distribution, and posterior ranges of all model parameters.

(7) "148; please state the justification for the lifespan definition. It doesn't make sense to me. T appears in the bounds of the summation and, I think, in the term itself. Couldn't understand this."

Response: Thank you. We have added additional information to this subsection:

"To measure the effective life of antibiotics, we note that the consumption of Drug M is inversely related to the effective lifespan of Drugs A and B. If resistance to Drug A and B rises quickly, implying a short effective lifespan for these drugs, all future cases of gonorrhea will be treated with Drug M. We therefore defined the effective lifespan of Drugs A and B as the area under the curve of the annual percentage of gonorrhea cases that are successfully treated with Drugs A or B over 50 years of simulation (i.e. $\sum_{t=1}^{50} \frac{N_A(t)+N_B(t)}{N_A(t)+N_B(t)+N_M(t)}$, where $N_A(t)$, $N_B(t)$ and $N_M(t)$ are the number of gonorrhea cases treated successfully with Drugs A, B, or M in simulation year t."

(8) "Fig 3: See comments about the lifespan definition. I couldn't understand what this was telling me at this point. Is the y axis absolute or some kind of relative percentage. If absolute, its tricky to interpret because I have to look back at the first figure."

Response: Please see our response to comment 7 above. The y-axes in Fig 3 show the change in annual number of cases and the x-axes shows the change in the effective lifespan of Drug A and B.

(9) “168: So Fig 3 is per 100,000. COmparing this with Fug 2b, they seem to be very modest changes?”

Response: Please note that the blue curves (labeled as ‘Threshold’) are identical in all panels of Fig 3.

(10) “180; maybe a discussion of the accuracy with which the trend would need to be known is important. If not here then later. Accurately measuring small changes would not be wihtout costs.”

Response: We agree with the Reviewer that the performance of strategies that use the estimated change in resistance prevalence to inform first-line treatment recommendations depends on the accuracy of estimated change. To demonstrate this, we have considered the strategy ‘Enhanced Threshold+Trend’ which relies on a larger number of isolates tested for drug-susceptibility when estimating the prevalence and the change in the prevalence of resistance. Also, we have discussed the cost of enhancing the surveillance in the Discussion section.

(11) “202; this paragraph is a simple reststement of results. It doesn't quite make sense here in the discussion.”

Response: Here, we followed the instructions provided in Submission Guidelines (https://journals.plos.org/plosmedicine/s/submission-guidelines#loc-discussion) that summary of the article's findings should be included in the Discussion section. We are happy to revise as the editors and reviewers see fit.

(12) “217; I'm pretty sure that the UK policy was influenced by the work by whittles et al in this journal that inferred fitness costs for specific drugs.”

Response: Thank you. Please see our response to comment 2.
